# Association Between Early-Life Exposure to Antibiotics and Development of Child Obesity: Population-Based Study in Italy

**DOI:** 10.2196/51734

**Published:** 2024-05-31

**Authors:** Anna Cantarutti, Paola Rescigno, Claudia Da Borso, Joaquin Gutierrez de Rubalcava Doblas, Silvia Bressan, Elisa Barbieri, Carlo Giaquinto, Cristina Canova

**Affiliations:** 1 Department of Statistics and Quantitative Methods University of Milano-Bicocca Milano Italy; 2 Department of Cardio-Thoraco-Vascular Sciences and Public Health University of Padova Padova Italy; 3 Department of Women’s and Children’s Health University of Padova Padova Italy

**Keywords:** childhood obesity, BMI z score, pediatric population-based, antibiotics, real-world data, association, exposure, child obesity, obesity, population-based, gut microbiome, early life, pediatric, prescription

## Abstract

**Background:**

Childhood obesity is a significant public health problem representing the most severe challenge in the world. Antibiotic exposure in early life has been identified as a potential factor that can disrupt the development of the gut microbiome, which may have implications for obesity.

**Objective:**

This study aims to evaluate the risk of developing obesity among children exposed to antibiotics early in life.

**Methods:**

An Italian retrospective pediatric population-based cohort study of children born between 2004 and 2018 was adopted using the Pedianet database. Children were required to be born at term, with normal weight, and without genetic diseases or congenital anomalies. We assessed the timing of the first antibiotic prescription from birth to 6, 12, and 24 months of life and the dose-response relationship via the number of antibiotic prescriptions recorded in the first year of life (none, 1, 2, and ≥3 prescriptions). Obesity was defined as a BMI *z* score >3 for children aged ≤5 years and >2 for children aged >5 years, using the World Health Organization growth references. The obese incidence rate (IR) × 100 person-years and the relative 95% CI were computed using infant sex, area of residence, preschool and school age, and area deprivation index, which are the covariates of interest. A mixed-effect Cox proportional hazards model was used to estimate the hazard ratio and 95% CI for the association between antibiotic exposure in early life and child obesity between 24 months and 14 years of age, considering the family pediatricians as a random factor. Several subgroup and sensitivity analyses were performed to assess the robustness of our results.

**Results:**

Among 121,540 children identified, 54,698 were prescribed at least an antibiotic within the first year of life and 26,990 were classified as obese during follow-up with an incidence rate of 4.05 cases (95% CI 4.01-4.10) × 100 person-year. The risk of obesity remained consistent across different timings of antibiotic prescriptions at 6 months, 1 year, and 2 years (fully adjusted hazard ratio [aHR] 1.07, 95% CI 1.04-1.10; aHR 1.06, 95% CI 1.03-1.09; and aHR 1.07, 95% CI 1.04-1.10, respectively). Increasing the number of antibiotic exposures increases the risk of obesity significantly (*P* trend<.001). The individual-specific age analysis showed that starting antibiotic therapy very early (between 0 and 5 months) had the greatest impact (aHR 1.12, 95% CI 1.08-1.17) on childhood obesity with respect to what was observed among those who were first prescribed antibiotics after the fifth month of life. These results were consistent across subgroup and sensitivity analyses.

**Conclusions:**

The results from this large population-based study support the association between early exposure to antibiotics and an increased risk of childhood obesity. This association becomes progressively stronger with both increasing numbers of antibiotic prescriptions and younger age at the time of the first prescription.

## Introduction

Childhood obesity is a significant public health problem representing the most severe challenge in the world that can have long-term consequences [1]. In the United States, 1 in 3 children and adolescents are overweight or obese [2]. In Europe, the prevalence has been increasing over the past few decades. According to the World Health Organization (WHO) and the European Childhood Obesity Surveillance Initiative, 38% of Italian children were found to be overweight (including obesity), with boys more affected than girls (41% and 35%, respectively). Moreover, the overall prevalence of obesity in Italy was 16% and more common in boys (20%) than girls (13%) [3].

Obesity is a chronic and complex disease characterized by excessive adiposity that can impair health, leading to various health complications, including an increased risk of developing chronic conditions such as type 2 diabetes, cardiovascular diseases, and mental health issues. It can also have social and emotional consequences, such as low self-esteem and stigmatization [4]. Considering these dramatic consequences, it is crucial to implement interventions to prevent the occurrence of this condition in children, which is also the goal of Health4EUkids, the European Joint Action for the implementation of Best Practices for the promotion of health and the prevention of obesity [5].

Early childhood obesity has been associated with several factors, such as maternal prepregnancy BMI, nutritional intake, physical activity, sleep duration, and screen time [6]. In particular, sleep deprivation can disrupt hormones that regulate appetite and metabolism, and excessive screen time can contribute to early childhood obesity by reducing physical activity and promoting unhealthy eating habits. In addition, emerging evidence suggests that the composition and function of the gut microbiome can influence various aspects of human health, including energy metabolism and body weight regulation. Previous studies demonstrated that the intestinal microbiome plays an important role in host energy metabolism, including gene expression that impacts energy availability from short-chain fatty acids and the processing of otherwise indigestible polysaccharides [2]. The microbial ecosystem begins taxonomic diversification at birth and completes its development during the early years of life [7]. The establishment and maturation of the gut microbiome are influenced by a complex interplay of internal and external factors, including environmental factors, the type of delivery (natural birth or cesarean delivery), and diet. While the gut microbiome is relatively stable, it can experience periods of acute or chronic perturbation in certain disease states or due to new exposures such as antibiotic therapies [8]. Antibiotic exposure in early life has been identified as a potential factor that can disrupt the development and composition of the gut microbiome, which may have implications for obesity later in life [7]. Previous epidemiological studies have provided valuable insights into the relationship between the gut microbiome, antibiotic exposure in early life, and obesity [2]. Early-life antibiotic exposures can modify the bacterial diversity of the intestinal microbiome in infants and delay microbiota maturation. These effects were most pronounced with antibiotic exposure during the first year of life, while no significant effect was observed with later exposures [9]. However, it is important to note that other confounding factors, such as genetic predisposition, diet, lifestyle, and socioeconomic factors, could contribute to both antibiotic use and obesity risk. Furthermore, the specific antibiotics used, the timing and duration of exposure, and the individual’s age at the time of exposure may influence the observed associations.

The objective of this study is to examine the risk of developing obesity among children exposed to antibiotics early in life in a large population-based Italian birth cohort with a detailed assessment of antibiotic use and long-term follow-up to assess the development of obesity identified through standardized anthropometric measurements.

## Methods

### Study Population

We used data from Pedianet [10], an independent network of more than 400 family pediatricians (FPs) established in 1998, to collect information from outpatient routine clinical care in Italy; detailed information was explained elsewhere [11]. In particular, in this study, we used information regarding demographic data, prescriptions (pharmaceutical prescriptions identified by the Anatomical Therapeutical Chemical code), and growth parameters. In Italy, FPs have been considered by the Ministry of Health as physicians responsible for performing regular “mandatory” well-child visits for preventive medicine purposes at specific time points during which the child’s anthropometric measurements are recorded [12].

We identified all children born between 2004 and 2018, followed from birth to at least 4 years (maximum follow-up of 14 years). Children were required to have at least 2 visits during the first 2 years of life at least 6 months apart [13], born at term (≥37 gestational weeks) and with birth weight greater than 2500 g, and without genetic diseases (ie, achondroplasia, Cornelia de Lange, Down, Prader-Willi, Turner, and Williams syndromes [14-21]) or congenital anomalies. The final cohort consisted of 121,540 children (Figure S1 in Multimedia Appendix 1).

### Ethical Considerations

This is an observational, retrospective, noninterventional study. According to a bylaw on the classification and implementation of observational drug-related research, as issued by the Italian National Drug Agency (an entity belonging to the Italian Ministry of Health), this study does not require approval by an ethics committee in Italy (Italian Drug Agency note on August 3, 2007). This study was conducted in accordance with the tenets of the Declaration of Helsinki and was compliant with the European Network of Centres for Pharmacoepidemiology and Pharmacovigilance’s Guide on Methodological Standards in Pharmacoepidemiology. Data generated during routine patient care were collected and handled anonymously, in compliance with Italian regulations, and stored under a unique numerical identifier. Ethical approval of the study and access to the database was approved by the Internal Scientific Committee of So.Se.Te. Srl, the legal owner of Pedianet.

### Infant Antibiotic Exposure Assessment

Antibiotic exposure (Anatomical Therapeutical Chemical code: J01*) was assessed using the outpatient prescription data recorded during the primary care visits. We assessed (1) the timing of the first antibiotic prescription from birth to 6, 12 (ie, the primary exposure of interest), and 24 months of life (Table S1 in Multimedia Appendix 1) and (2) the dose-response relationship via the number of antibiotic prescriptions recorded in the first year of life (none, 1, 2, and ≥3 prescriptions). Moreover, specific classes of antibiotics were categorized based on the spectrum of action (ie, narrow and broad-spectrum antibiotics) and independent assessment (ie, penicillins, cephalosporins, macrolides, and others; Table S2 in Multimedia Appendix 1) [22].

### Child Obesity Assessment

Data for weight and height were retrieved from the growth parameters recorded during the primary care visits and ascertained longitudinally from 2 years until the last parameter recorded at the end of the study or the completion of the 14th year of life. BMI was calculated as weight (kg)/length (m^2^) and transformed into age- and sex-specific *z* score using the WHO Growth References [23-25]. Obesity was defined as a *z* score >3 SDs above the mean for children aged ≤5 years and a *z* score >2 SDs for children aged >5 years [26]. Children without growth parameters recorded from the second birthday or with implausible BMI *z* score values (ie, *z* score <4 and *z* score >8) were excluded from the analysis [27]. We also performed a sensitivity analysis where obesity was redefined based on the Centers for Disease Control and Prevention (CDC) Growth Charts, identifying children as obese for values of the BMI *z* score >2 [28].

### Covariates

Additional factors such as the birth year, infant sex, area of residence (ie, north, center, south, and islands of Italy), the area deprivation index (ADI 1—least deprived; ADI 2, 3, 4, 5—most deprived; and ADI missing [29]), and the FP were considered of interest. The ADI is based on 5 items that recurrently describe social and material deprivation and is categorized in quintiles based on the regional ADI level to ensure within-region appropriately represented categories.

### Statistical Analysis

Demographic characteristics were summarized through frequency and percentages and compared obese with nonobese children. The chi-square test was used to assess differences. Moreover, the obese incidence rate (IR) per 100 person-year (PY) and the relative 95% CI were computed using infant sex, area of residence, preschool (ie, obese within ≤5 years of age), school-aged children (ie, obese over >5 years of age), and ADI.

A mixed-effect Cox proportional hazards model was used to estimate the hazard ratio and 95% CI for the association between antibiotic exposure within the first year of life (exposure at 1 year) and child obesity between 24 months and 14 years of age, considering the FPs as a random factor. The proportional hazard assumption for the time-fixed covariates was tested using Schoenfeld residuals [30]. The follow-up began after 2 years of age and ended with the last anthropometric measure available at the end of the study (ie, December 31, 2022), the completion of the 14th year of life, or the end of pediatric assistance. We performed 2 levels of adjustment, including (1) infant sex, area of residence, and year of birth, and (2) infant sex, area of residence, year of birth, and ADI as categorical variables (considering missing as the sixth category).

In addition, we evaluated the association between (1) the timing of the first antibiotic prescription from birth to 6 (exposure at 6 months) and 24 (exposure at 2 years) months, (2) the dose-response relationship by assessing the number of antibiotic prescriptions recorded at 1 year, and (3) the individual-specific age of the antibiotic therapy initiation by assessing the age at the first prescription within 1 year (0-<5, 5-<8, and 8-12 months) and the development of childhood obesity. The SAS software (version 9.4; SAS Institute) and R (R Foundation for Statistical Computing) were used for the analyses. For all hypotheses tested, 2-tailed *P*<.05 were considered to be significant.

### Subgroup and Sensitivity Analyses

We also performed several subgroup analyses. We assessed the association between child obesity with (1) the type of antibiotics prescribed in the first year based on the spectrum of action (ie, narrow and broad-spectrum antibiotics vs none) and the antibiotic class (ie, penicillins, cephalosporins, macrolides, and others vs none), (2) the preschool and school age children, and (3) the infant sex.

We performed several sensitivity analyses to examine the robustness of our findings. We repeated the main analysis (1) including only children with a birth weight within the normal range (ie, not exceeding 4000 g; n=4143, 3.4% excluded), (2) excluding from the cohort those children with missing values for the ADI (n=16,582, 13.6% excluded), (3) restricting the cohort to only those children resident in the Veneto region since it is the most representative region of Italy in Pedianet (n=49,772, 41% included), and (4) redefining the outcome variable calculating the BMI *z* score using the CDC Growth Charts.

## Results

### Description of the Cohort

Among 121,540 children identified in the Pedianet network within birth, 45% (54,698/121,540) were prescribed at least 1 antibiotic within the first year of life. The median follow-up period was approximately 6 (IQR 5-10) years.

Of the overall cohort, 22% (26,990/121,540) were classified as obese during follow-up, with an IR of 4.05 cases (95% CI 4.01-4.10) × 100 PY (Table 1). The IR of obesity was quite similar among male and female participants (IR 3.96 vs 4.16 × 100 PY, respectively), more than double in school-aged than preschool-aged children (IR 3.68 vs 1.48 × 100 PY, respectively), and higher in children from the south and islands (IR 3.56, 4.01, and 5.16 × 100 PY, respectively for children from north, center, and south of Italy). Moreover, the incidence of obesity increased with increasing deprivation index (IR 3.69 vs 4.23×100 PY for least and most deprived children, respectively; Table 1).

Among children without antibiotic prescription during their first year of life, 21% (13,956/66,842) were observed to be obese, while 24% (13,034/54,698) of those with antibiotic exposure were obese, with an IR of 3.82 × 100 PY (95% CI 3.76-3.89) and 4.34 × 100 PY (95% CI 4.26-4.41), respectively. The incidence of obesity was higher among antibiotic-exposed children than unexposed children across all baseline characteristics considered (Table 2; Table S3 in Multimedia Appendix 1).

**Table 1:** Baseline characteristics of children within the cohort and incidence rate of obesity among characteristics of interest (Pedianet, Italy; 2004-2018; N=121,540).

			Value, n (%)		IR^a^ × 100 PY^b^ (95% CI)
Obese—WHO^c^ definition			26,990 (22.21)		4.05 (4.01-4.1)
Obese—CDC^d^ definition			12,676 (10.43)		1.86 (1.83-1.89)
**Sex**					
	Male	63,025 (51.86)		3.96 (3.89-4.02)	
	Female	58,515 (48.14)		4.16 (4.09-4.23)	
**Year of birth**					
	2004-2007	35,248 (29)		—^e^	
	2008-2011	39,237 (32.28)		—	
	2012-2017	47,055 (38.72)		—	
**Obesity age**					
	Preschool aged	—		1.48 (1.44-1.52)	
	School aged	—		3.68 (3.63-3.73)	
**Local area of residence**					
	North	69,661 (57.32)		3.56 (3.5-3.62)	
	Center	18,037 (14.84)		4.01 (3.9-4.13)	
	South and Islands	33,842 (27.84)		5.13 (5.03-5.24)	
**Deprivation index**					
	Missing	16,582 (13.64)		4.19 (4.06-4.33)	
	Low	20,546 (16.90)		3.69 (3.58-3.8)	
	Medium low	21,666 (17.83)		3.93 (3.82-4.04)	
	Medium	22,695 (18.67)		4.13 (4.01-4.24)	
	Medium high	20,979 (17.26)		4.22 (4.1-4.34)	
	High	19,072 (15.69)		4.23 (4.1-4.36)	

^a^IR: incidence rate.

^b^PY: person-year.

^c^WHO: World Health Organization.

^d^CDC: Centers for Disease Control and Prevention.

^e^Not applicable.

**Table 2:** IR^a^ and 95% CI of obesity among characteristics of interest by exposure groups (Pedianet, Italy; 2004-2018; N=121,540).

			IR × 100 PY^b^ (95% CI)		
			Unexposed children (n=66,842)		Antibiotic-exposed children (n=54,698)
**Sex**					
	Male	3.76 (3.67-3.85)		4.18 (4.08-4.28)	
	Female	3.88 (3.79-3.97)		4.52 (4.41-4.63)	
**Obesity age**					
	Preschool aged	1.32 (1.27-1.37)		1.67 (1.61-1.73)	
	School aged	3.53 (3.46-3.59)		3.86 (3.79-3.94)	
**Local area of residence**					
	North	3.44 (3.37-3.52)		3.76 (3.66-3.86)	
	Center	4.05 (3.89-4.22)		3.97 (3.8-4.14)	
	South and Islands	4.83 (4.67-4.99)		5.36 (5.22-5.5)	
**Deprivation index**					
	Missing	3.87 (3.69-4.05)		4.58 (4.36-4.79)	
	Low	3.47 (3.33-3.62)		3.97 (3.8-4.15)	
	Medium low	3.72 (3.58-3.87)		4.19 (4.02-4.37)	
	Medium	3.93 (3.78-4.08)		4.36 (4.19-4.53)	
	Medium high	3.99 (3.83-4.15)		4.5 (4.32-4.68)	
	High	4.02 (3.85-4.19)		4.47 (4.27-4.66)	

^a^IR: incidence rate.

^b^PY: person-year.

### Association Between Early Antibiotic Exposure and Incidence of Obesity

The findings presented in Table 3 are directly relevant to the central goal of this research investigation. The risk of obesity remained consistent across different timing of antibiotic prescriptions at 6 months, 1 year, and 2 years (fully adjusted HR [aHR] 1.07, 95% CI 1.04-1.10; aHR 1.06, 95% CI 1.03-1.09; aHR 1.07, 95% CI 1.04-1.10, respectively). No significant differences were observed between partially and fully adjusted analyses, even when restricting the cohort to complete cases for the ADI. Increasing the number of antibiotic exposures increases the risk of obesity significantly (*P* for trend<.001). Compared to children with no antibiotic prescriptions, those with 1, 2, or ≥3 prescriptions had an increased risk of 4% (95% CI 1-7), 6% (95% CI 2-10), and 14% (95% CI 9-18), respectively. The individual-specific age analysis showed that starting antibiotic therapy very early (between 0 and 5 months) had the greatest impact (aHR 1.12, 95% CI 1.08-1.17) on childhood obesity with respect to what was observed among those who were first prescribed antibiotics between 5 and 8 months of life (aHR 1.08, 95% CI 1.04-1.12) and the 8-12 months of life (aHR 1.03, 95% CI 0.99-1.06).

Subgroup analyses showed a higher risk of obesity among preschool-aged children (aHR 1.11, 95% CI 1.05-1.18) than school-aged children (aHR 1.05, 95% CI 1.02-1.09). When examining the subgroup of male and female participants separately, antibiotic exposure in the first year of life was associated with an increased risk of obesity of 4% (95% CI 1%-8%) and 10% (95% CI 7%-15%), respectively. However, the difference between female and male participants in the risk of obesity was not significant (*P*=.11). The increased risk of obesity among exposed children was consistent when analyzing only children with a birth weight within the normal range (aHR 1.06, 95% CI 1.03-1.09) and from the Veneto Region (aHR 1.07, 95% CI 1.02-1.11). In addition, using the CDC definition of obesity showed results consistent with the WHO definition (aHR 1.08, 95% CI 1.04-1.13).

Table 4 presents analyses based on the spectrum of action and class of antibiotic in the first year of life. Exposure to narrow-spectrum antibiotics appears associated with a higher risk of obesity (aHR 1.09, 95% CI 1.06-1.12) compared to broad-spectrum antibiotics (aHR 1.06, 95% CI 1.02-1.09). However, the difference between narrow- and broad-spectrum antibiotics in the risk of obesity was not significant (*P*=.52). In addition, the class of macrolides showed a stronger association with the risk of obesity (aHR 1.13, 95% CI 1.08-1.17), followed by other categories (aHR 1.10, 95% CI 1.00-1.20), while penicillins (aHR 1.07, 95% CI 1.04-1.10) and cephalosporins (aHR 1.07, 95% CI 1.03-1.12) had a similar extent of association.

**Table 3:** Partially and fully adjusted models assessing the association between antibiotic exposure and the risk of obesity (Pedianet, Italy; 2004-2018; N=121,540).

Characteristics								Children, n			Children exposed to antibiotics, n			Obese children exposed to antibiotics, n			HR^a^ (95% CI)			*P* for trend	
**Main analysis**																					
	**Exposure at 1 year**						121,540			54,698			13,034						—^b^		
		Partially adjusted														1.06 (1.03-1.09)					
		Fully adjusted														1.06 (1.03-1.09)					
		**Complete case analysis^c^**					104,958			47,284			11,226								
				Partially adjusted											1.06 (1.03-1.09)						
				Fully adjusted											1.06 (1.03-1.09)						
	**Dose-response analysis (** **fully adjusted** **)**																				
			**Exposure at 1 year**				121,540												<.001		
					None				66,842			13,956			1.00 (Reference)						
					1 RX^d^				26,619			5856			1.04 (1.01-1.07)						
					2 RXs				13,401			3175			1.06 (1.02-1.10)						
					≥3 RXs				14,678			4003			1.14 (1.09-1.18)						
	**Individual-specific age for exposure at 1 year**																			.03	
					None	121,540			66,842			13,956			1.00 (Reference)						
					≥0 months and <5 months	121,540			15,020			3849			1.12 (1.08-1.17)						
					≥5 months and <8 months	121,540			16,542			3950			1.08 (1.04-1.12)						
					≥8 months and ≤12 months	121,540			23,136			5235			1.03 (0.99-1.06)						
			Exposure at 6 months (fully adjusted)				121,540			23,576			5889			1.07 (1.04-1.10)					
			Exposure at 2 years (fully adjusted)				121,540			83,846			19,405			1.07 (1.04-1.10)					
**Subgroup analysis**																					—
	Normal birth weight (fully adjusted)						117,397			52,543			12,409			1.06 (1.03-1.09)					
	Preschool aged (fully adjusted)						121,540			5986			3046			1.11 (1.05-1.18)					
	School-aged (fully adjusted)						77,537			21,004			9988			1.05 (1.02-1.09)					
	Female (fully adjusted)						58,515			25,210			6254			1.10 (1.07-1.15)					
	Male (fully adjusted)						63,025			29,488			6780			1.04 (1.01-1.08)					
	Veneto region (fully adjusted)						49,772			9518			3826			1.07 (1.02-1.11)					
	CDC^e^ definition (fully adjusted)						121,553			12,676			6413			1.08 (1.04-1.13)					

^a^HR: hazard ratio.

^b^Not applicable.

^c^The complete case analysis included 104,858 children.

^d^RX: prescription.

^e^CDC: Centers for Disease Control and Prevention.

**Table 4:** Fully adjusted models assessing the association between the spectrum of action and class of antibiotic therapy and the risk of obesity (Pedianet, Italy; 2004-2018; N=121,540).

		Children, n	Children exposed to antibiotics, n	Obese children exposed to antibiotics, n	HR^a^ (95% CI)
**Spectrum of action**					
	Narrow-spectrum antibiotics	99,170	32,328	7856	1.09 (1.06-1.12)
	Broad-spectrum antibiotics	101,844	35,002	8503	1.06 (1.02-1.09)
**Class of antibiotic therapy**					
	Penicillins	107,516	35,295	8128	1.07 (1.04-1.10)
	Cephalosporins	81,356	12,355	3279	1.07 (1.03-1.12)
	Macrolides	81,989	13,042	3555	1.13 (1.08-1.17)
	Others	69,333	2110	510	1.10 (1.00-1.20)

^a^HR: hazard ratio.

## Discussion

### Principal Findings

In this pediatric population–based cohort study of 121,540 children, we found a 6% (aHR 1.06, 95% CI 1.03-1.09) increased risk of developing childhood obesity among children exposed to antibiotics within the first year of life compared to unexposed children. This relationship is stronger as the number of prescriptions increases and as the individual’s age at the first prescription of antibiotics decreases. The results were consistent across all sensitivity and subgroup analyses conducted.

Obesity is a significant public health concern worldwide, especially among children and young people. Italy is one of the countries in Europe with the highest rate of childhood obesity: according to the latest report by “OKkio alla Salute” from the Ministry of Health in 2019, 38% are overweight and 16.5% are obese among school-aged children; they also used the WHO Growth References [31]. There continued to be a large difference between countries. Overweight prevalence in children varied remarkably from 6% in Tajikistan to 43% in Cyprus. Similarly, obesity rates spanned a wide spectrum, from a minimal 1% in Tajikistan to a concerning 19% in Cyprus. In our cohort, 22% of children were classified as obese at least in 1 measurement during the follow-up when the age- and sex-specific *z* score was calculated using the WHO Growth References, with respect to 10% when the CDC Growth Charts were used. The reason for this discrepancy relies on the fact that while the CDC 2000 Growth Charts represent the reference growth charts for the US pediatric population, the WHO 2006 ones are intended to serve as growth chart standards, describing how children should grow globally and not how they did grow in a specific nation. Indeed, while the CDC Growth Charts were developed from 5 nationally representative survey data sets from the United States (the National Health Examination Surveys), the WHO ones were based on data from a Multicenter Growth Reference Study that collected a highly selective sample of children from 6 sites around the world (Brazil, Ghana, India, Norway, Oman, and the United States), consisting of children who were not subjected to socioeconomic constraints on growth, who were fed according to the study feeding recommendations, who were healthy term singleton births, and whose mothers did not smoke; the growth of these children was considered to represent optimal growth. Several studies have subsequently observed a significant difference in the rate of overweight or underweight children depending on the growth charts used in national prevalence studies [32-34].

Previous studies have investigated the association between early antibiotic exposure and childhood obesity and reported inconsistent results [2,29-33]. Our results confirm previous findings demonstrating a positive association through several exhaustive analyses, even with attenuated estimations [35-39]. Moreover, our results confirmed previous evidence that the risk of childhood obesity was more significant in those children who received more than 3 antibiotics in the early years of life [37] and with earlier start-exposure timing [39]. However, our results did not support previous evidence showing stronger associations between obesity risk and the male sex of the children. In fact, we found an increased risk of obesity among female participants (aHR 1.10, 95% CI 1.07-1.15) compared with male participants (aHR 1.04, 95% CI 1.01-1.08) [39].

Furthermore, we explored the impact of different spectrums of action and types of antibiotics on childhood obesity. Conversely to Bailey et al [2] who found a stronger association for broad-spectrum antibiotics but not for narrow-spectrum therapy, our findings revealed a positive association with both narrow- and broad-spectrum antibiotics.

The causes of childhood obesity are complex and multifactorial, although the hypothesis that gut microbiota plays a crucial role in the pathogenesis of obesity is well established [40-43]. Recent studies have indicated that antibiotic use leads to alterations in the gut microbiota, potentially affecting nutrient absorption and resulting in metabolic imbalances that contribute to obesity [7]. Our results corroborate what has already been shown in various laboratory models, supporting the notion that weight gain induced by antibiotics is mediated through the drug’s impact on the gut microbiome and the consequently altered circulating levels of substances (eg, short-chain fatty acids, secondary biliary acids, and branched and aromatic amino acids) that influence human metabolism [7]. Changes in microbiota caused by antibiotic use are defined as dysbiosis, a microbial imbalance correlated with impaired health, including increased susceptibility to infections, impaired immune function, gastrointestinal symptoms, and even long-term effects on metabolic health and obesity [44]. Obesity is a chronic and complex disease with significant long-term effects; hence, it is crucial to implement interventions to prevent this condition in children. Preventing and addressing childhood obesity require a comprehensive, multisectoral approach involving individuals, families, communities, educational institutions, health care systems, and policy makers. Strategies may include promoting healthy eating habits, increasing physical activity opportunities, improving food environments, and implementing policies that restrict the marketing of unhealthy foods to children as also supported by the Health4EUkids project; the European Joint Action for the Implementation of Best Practices for the promotion of health and the prevention of obesity [5].

There are several potential limitations to consider in this research. First, exposure to antibiotics was defined by prescription, assuming that a prescription led to actual medication use. However, this assumption may not always hold true, leading to possible misclassification of the exposure. Anyhow, this misclassification is likely to bias the results toward the null hypothesis. Moreover, antibiotics prescribed in a private setting or hospitals are not recorded. Second, important confounding factors related to both pregnancy and early life were not captured in Pedianet. This includes factors like maternal BMI before and during pregnancy, breastfeeding, method of complementary feeding, dietary patterns, sugar-sweetened beverages consumption, eating behavior (eg, skipping breakfast and family dinners), meal frequency and composition (fast foods and snacking), portion size, physical activity, screen media exposure, and sedentary behavior [45,46]. These factors could potentially influence the results and lead to residual confounding. We adjusted the models for various sociodemographic characteristics to address some of these limitations. Notably, we included the area-level socioeconomic deprivation index, which is commonly used in public health research. It serves to quantify the extent of geographically determined social inequalities in health or assess the independent effect that area characteristics have on health beyond individual socioeconomic position. In addition, this index can help substitute missing individual-level data in epidemiological studies and account for confounding socioeconomic factors.

This study also has several strong points. First, using a pediatric population–based registry allowed for a large unbiased cohort with extended follow-up, thereby minimizing selection and recall bias. Second, to ensure accuracy and avoid potential exposure or outcome misclassification, we included only children who were consistently monitored by their FP during the first 2 years of life and had at least 1 reliable BMI measurement after completing the second year of life. Furthermore, setting the end of follow-up as the date of the last BMI measurement for each child helped minimize outcome misclassification. Finally, we used all the anthropometric measures (N=564,066) recorded during the pediatric visits performed by children included in the cohort. To our knowledge, no previous study has been conducted with this level of comprehensiveness and detail in terms of anthropometric data collection.

### Conclusions

In conclusion, the results from this large population-based study support the association between early exposure to antibiotics and an increased risk of childhood obesity. This relationship becomes more pronounced as the number of prescriptions increases and as the age specific for the first prescription of antibiotics decreases.

## Appendix

Multimedia Appendix 1 Supplementary material.

## Abbreviations

ADI: area deprivation index
aHR: adjusted hazard ratio
CDC: Centers for Disease Control and Prevention
FP: family pediatrician
IR: incidence rate
PY: person-year
WHO: World Health Organization

## Data Availability

The data supporting this study’s findings are available on request from the corresponding author (AC). The data are not publicly available due to restrictions (containing information that could compromise the privacy of research participants).
