# Supplementary material for: Association Between Early-Life Exposure to Antibiotics and Development of Child Obesity: Population-Based Study in Italy
Source: JMIR Public Health Surveill. 2024 May 31;10:e51734. doi: 10.2196/51734 (PMC11179038; doi:10.2196/51734)
Supplement: Multimedia Appendix 1 [file publichealth_v10i1e51734_app1.docx]

**Multimedia Appendix 1**

The authors have provided this appendix to give readers additional information about their work. Pedianet, 2004-2018, N=121,540.

Figure S1. Flow-chart


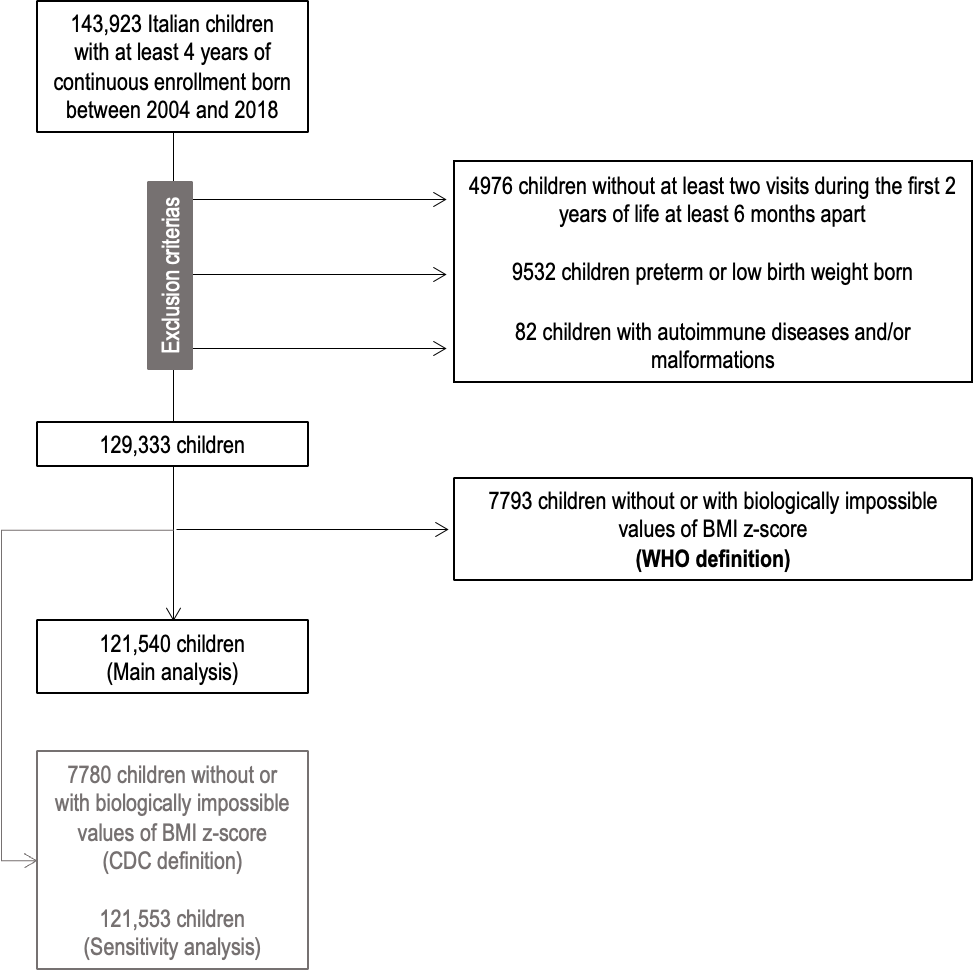


| Table S1. Distribution of timing of exposure. Pedianet, 2004-2018, N=121,540. | | | | |
| --- | --- | --- | --- | --- |
| Exposure 6 mo. | Exposure 1 yr. | Exposure 2 yr. | N | (%) |
|  |  |  | 37694 | (31) |
|  |  |  | 29148 | (24) |
|  |  |  | 31122 | (26) |
|  |  |  | 23576 | (19) |

| Table S2. ATC codes for classification based on the Spectrum of action and Class of antibiotic therapy | |
| --- | --- |
| Spectrum of action | ATC codes |
| Narrow-spectrum antibiotics | J01CA04, J01CR04, J01DB01, J01FA06, J01FA09, J01FF02, J01XE01, J01XX01, J01XX03 |
| Broad-spectrum antibiotics | J01CA01, J01CR01, J01CR02, J01DC02, J01DC04, J01DC06, J01DC10, J01DD01, J01DD02, J01DD04, J01DD08, J01DD13, J01DD14, J01DE01, J01FA01, J01FA02, J01FA07, J01FA10, J01FA11, J01FA12 |
|  |  |
| Class of antibiotic therapy |  |
| Penicillins | J01CA04, J01CA01, J01CR01, J01CR02, J01CR04 |
| Cephalosporins | J01DB01, J01DC02, J01DC04, J01DC06, J01DC10, J01DD01, J01DD02, J01DD04, J01DD08, J01DD13, J01DD14, J01DE01 |
| Macrolides | J01FA01, J01FA02, J01FA06, J01FA07, J01FA09, J01FA10, J01FA11, J01FA12 |
| Others | J01FF02, J01XE01, J01XX01, J01XX03 |

| Table S3. Incidence of obesity overall and among characteristics of interest. Pedianet, 2004-2018, N=121,540. | | | |
| --- | --- | --- | --- |
|  | N.ro of obese | Person-Years | I (95% CI) x 100 person-yr. |
| Overall Obese |  |  |  |
| WHO definition | 26990 | 665744.7 | 4.05 (4.01 - 4.1) |
| CDC Definition | 12676 | 681686.67 | 1.86 (1.83 - 1.89) |
|  |  |  |  |
| Infant sex |  |  |  |
| Male |  |  |  |
| Unexposed | 6958 | 184912.12 | 3.76 (3.67 - 3.85) |
| Exposed | 6780 | 162316.89 | 4.18 (4.08 - 4.28) |
| Female |  |  |  |
| Unexposed | 6998 | 180185 | 3.88 (3.79 - 3.97) |
| Exposed | 6254 | 138330.69 | 4.52 (4.41 - 4.63) |
| Local area of origin |  |  |  |
| North |  |  |  |
| Unexposed | 8035 | 233446.22 | 3.44 (3.37 - 3.52) |
| Exposed | 5525 | 147000.62 | 3.76 (3.66 - 3.86) |
| Center |  |  |  |
| Unexposed | 2288 | 56453.8 | 4.05 (3.89 - 4.22) |
| Exposed | 2082 | 52399.86 | 3.97 (3.8 - 4.14) |
| South & Islands |  |  |  |
| Unexposed | 3633 | 75197.09 | 4.83 (4.67 - 4.99) |
| Exposed | 5427 | 101247.11 | 5.36 (5.22 - 5.5) |
| Time |  |  |  |
| Pre-school aged |  |  |  |
| Unexposed | 2940 | 222259.14 | 1.32 (1.27 - 1.37) |
| Exposed | 3046 | 182550.74 | 1.67 (1.61 - 1.73) |
| School-aged |  |  |  |
| Unexposed | 11016 | 312489.98 | 3.53 (3.46 - 3.59) |
| Exposed | 9988 | 258592.85 | 3.86 (3.79 - 3.94) |
| Area-deprivation index |  |  |  |
| ADI 1 |  |  |  |
| Unexposed | 2345 | 67482.77 | 3.47 (3.33 - 3.62) |
| Exposed | 1979 | 49815.7 | 3.97 (3.8 - 4.15) |
| ADI 2 |  |  |  |
| Unexposed | 2514 | 67560.8 | 3.72 (3.58 - 3.87) |
| Exposed | 2275 | 54233.58 | 4.19 (4.02 - 4.37) |
| ADI 3 |  |  |  |
| Unexposed | 2657 | 67637.79 | 3.93 (3.78 - 4.08) |
| Exposed | 2507 | 57494.25 | 4.36 (4.19 - 4.53) |
| ADI 4 |  |  |  |
| Unexposed | 2467 | 61860.77 | 3.99 (3.83 - 4.15) |
| Exposed | 2371 | 52707.31 | 4.5 (4.32 - 4.68) |
| ADI 5 |  |  |  |
| Unexposed | 2155 | 53575.52 | 4.02 (3.85 - 4.19) |
| Exposed | 2094 | 46884.48 | 4.47 (4.27 - 4.66) |
| ADI Missing |  |  |  |
| Unexposed | 1818 | 46979.46 | 3.87 (3.69 - 4.05) |
| Exposed | 1808 | 39512.26 | 4.58 (4.36 - 4.79) |
